# The comparative effects of Cantonese and Mandarin tone language backgrounds on musical pitch perception

**DOI:** 10.1038/s41598-025-17171-2

**Published:** 2025-08-27

**Authors:** William Choi, Lok Yan Chan

**Affiliations:** 1 Academic Unit of Human Communication, Learning, and Development, The University of Hong Kong, Hong Kong, China; 2 Speech and Music Perception Laboratory, The University of Hong Kong, Hong Kong, China; 3 Centre for Advancement in Inclusive and Special Education, The University of Hong Kong, Hong Kong, China

**Keywords:** Music-to-language transfer, Pitch, Tone, Cantonese, Mandarin, Music perception, Psychology, Perception, Language

## Abstract

Existing studies on language-to-music language transfer suggest that tone language background enhances musical pitch perception. However, tone language background was underrepresented as a binary yes-or-no variable in these studies. To extend the previous studies, we investigated the comparative effects of Cantonese and Mandarin language backgrounds on musical pitch perception. Forty-eight native Cantonese and Mandarin listeners were tested on static musical pitch, musical pitch interval, and dynamic musical pitch discrimination. Overall, the Cantonese listeners outperformed the Mandarin listeners in terms of sensitivity index and accuracy but not response time. The results indicate that Cantonese listeners exhibit superior abilities in discriminating static and dynamic musical pitch compared to Mandarin listeners. From a theoretical perspective, these results provide nuanced evidence for language-to-music transfer, indicating that different tone language backgrounds may enhance musical pitch perception to differing degrees. Additionally, the findings support the bi-directional OPERA hypothesis and motivate future studies to theoretically account for language-to-music transfer.

## Introduction

Music and speech share various functional and acoustic commonalities^1^. Functionally, music and speech are communicative means through which humans express emotions and ideas. Acoustically, pitch is an important feature that defines the melody of music^2^. In speech, pitch conveys speaker emotion, aids speech segmentation, and can even distinguish words in some languages (e.g., lexical tones)^3–5^. Given these commonalities, researchers have extensively examined the positive effect of music training on speech and language abilities, known as *music-to-language transfer*^6–8^. In contrast, there has been little empirical attention on the opposite direction, i.e., *language-to-music transfer*. Previous research on this topic found that listeners who speak a tone language have better musical pitch perception compared to those who do not^9–11^. Critically, different tone languages have different lexical tone systems^5,12,13^, but the existing studies have often underrepresented tone language background as a binary yes-or-no variable. From a theoretical perspective, neglecting the diversity of lexical tone systems could result in an oversimplified view of language-to-music transfer, failing to capture its complex and nuanced nature. To address this research gap, the present study examined the comparative effects of Cantonese and Mandarin language backgrounds on musical pitch perception.

### Music-to-language transfer and the OPERA hypothesis

The OPERA hypothesis proposes the mechanism of music-to-language transfer in speech perception^14,15^. As the present study focuses on tone language background, we will illustrate OPERA with lexical tone perception. According to OPERA, music training can enhance lexical tone perception as long as five conditions are met, namely overlap, precision, emotion, repetition, and attention. “Overlap” means that the processing of their acoustic features (i.e., fundamental frequency or *F0*) recruits overlapping subcortical networks. For “precision” to be met, music must require more fine-grained F0 processing than speech. For example, a small deviation of just 1 semitone can lead to a salient out-of-key note in music, whereas lexical tones differ by 2 to 7 semitones on average^14,16^. In addition, the music training must elicit strong positive emotions, be repeated frequently, and involve focused attention. If the five conditions are met, music training would increase neuronal sensitivity to F0, in turn benefiting lexical tone perception.

In line with the OPERA framework, musicians were frequently found to excel in lexical tone perception relative to non-musicians^6,7,17^. In an early study, English musicians exhibited higher accuracy than English non-musicians in discriminating Mandarin tones^18^. Moreover, English musicians identified Mandarin tones more accurately than English non-musicians, and even performed on par with Mandarin listeners^17^. Similar findings were reported in Italian listeners^7^, Cantonese tones^6,19^, and Thai tones^20,21^. Taken together, these findings provide correlational evidence of the effect of music training on lexical tone perception. For causal evidence, a randomized controlled trial found that piano training increased Mandarin children’s neural sensitivity to lexical tone changes, as indexed by an increased amplitude of positive mismatch responses^8^.

Of interest to our study, OPERA does not seem to support the notion of language-to-music transfer^14,15,22^. Although Patel has not explicitly discussed about the bi-directionality of OPERA, lexical tone perception hardly requires more fine-grained F0 processing than musical pitch perception^14,16^. Thus, tone language background is unlikely to fulfill the “precision” condition. Besides “precision”, some propose that speaking a tone language may not fulfill the “emotion” condition as playing music does^23^. As meeting the five conditions is essential for cross-domain transfer, OPERA implies that speaking a tone language is unlikely to enhance musical pitch perception.

## Language-to-music transfer and the bi-directional OPERA hypothesis

Inconsistent with the implication of OPERA, evidence suggests language-to-music transfer in musical pitch perception^11,24,25^. Relative to non-tonal languages, tone languages place a heavier demand on F0 for lexical processing^26^. Compared with English listeners, Cantonese listeners more accurately detected out-of-key musical notes^11^. Moreover, Cantonese listeners outperformed English listeners on discriminating and memorizing melodic sequences^9^. In addition, Cantonese listeners discriminated melodic sequences more accurately than English listeners, even with non-verbal intelligence controlled^24^. Similar findings were reported in other tone languages^10,27,28^. For example, Thai listeners outdid English listeners in a one-note musical pitch discrimination task^28^. Moreover, Mandarin listeners detected musical pitch interval changes more accurately than English listeners^29^. Furthermore, Yoruba (an African tone language) listeners discriminated dynamic musical pitch more accurately than did English listeners^27^.

Motivated by the above findings, the bi-directional OPERA hypothesis was proposed to account for language-to-music transfer^24^. In the original OPERA, “precision” was domain-relative, in which music always prevailed language^14^. In the bi-directional OPERA, “precision” is re-referenced on listeners^24^. Relative to English listeners, Cantonese listeners experience a stronger demand on precise F0 processing in their language^26^. Such precision is transferrable across domain, enhancing musical pitch perception in Cantonese listeners. In addition to language-to-music transfer, the bi-directional OPERA also explains music-to-language transfer. Specifically, it posits that musicians engage in more precise F0 processing than non-musicians, and their enhanced F0 perception is transferrable to the language domain.

## Research gap and the current study

Critically, previous studies have largely underrepresented tone language background as a binary yes-or-no variable^11,24,27^. Theoretically, this could lead to an oversimplified view of language-to-music transfer—that tone language background facilitates musical pitch perception. While we believe this view is broadly true, different tone languages place different demands on F0 perception^30,31^ which may result in different degrees of language-to-music transfer. Therefore, considering the diversity of tone languages is essential for a deeper understanding of the intricate nature of language-to-music transfer.

**Fig. 1:**
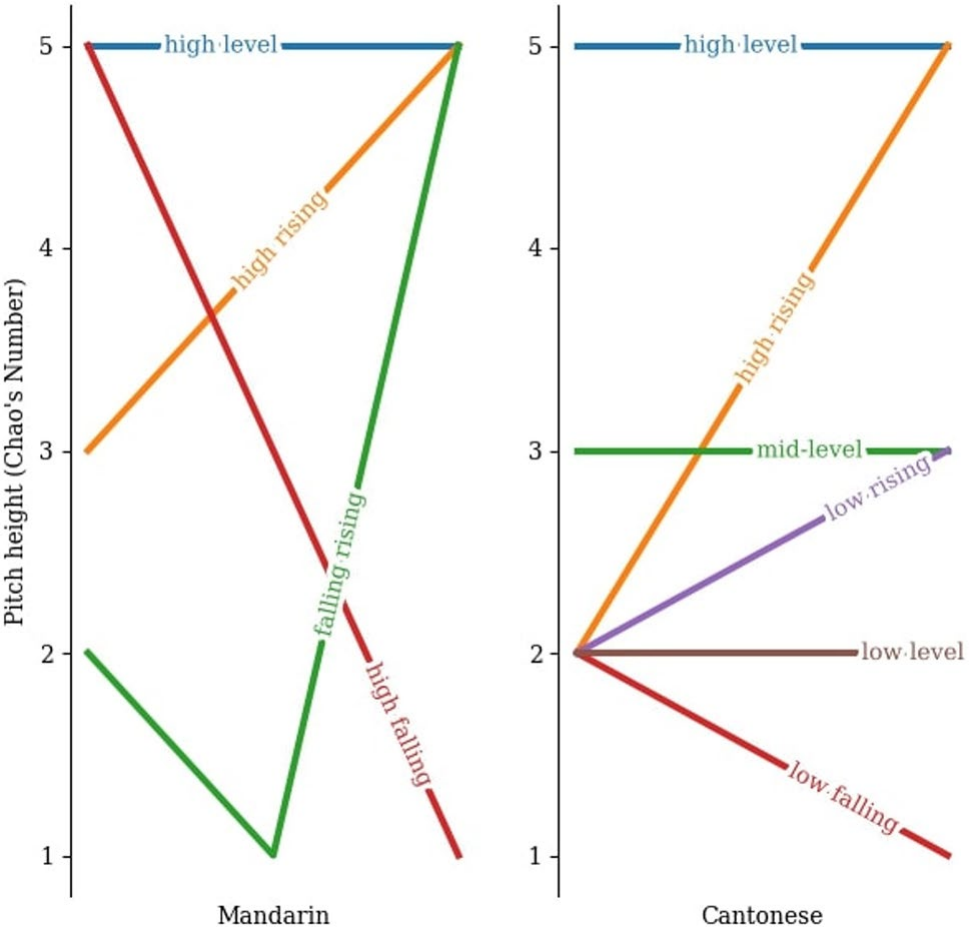
Mandarin and Cantonese tones transcribed by Chao^12^.

Of interest to us, Cantonese and Mandarin are both tone languages, but they differ in the complexity of their lexical tone systems^31,32^. Regarding the tonal inventory size, Mandarin has four lexical tones whereas Cantonese has six^5,12,33^. Based on subjective impression, Chao^12^ numerically transcribed the Mandarin and Cantonese lexical tones using a five-scale pitch system, in which 1 and 5 represent the lowest and highest pitches respectively. A level tone contains a stable pitch over time, whereas a contour tone involves relatively drastic pitch variations over time (e.g., falling, rising, and falling-rising)^5,12^. As illustrated in Fig. 1, Mandarin has one level tone (i.e., high level [55]) and three contour tones (i.e., high rising [35], falling rising [215], and high falling [51]). In contrast, Cantonese has three level tones (high level [55], mid-level [33], and low level [22]) and three contour tones (high rising [25], low falling [21], and low rising [23]). These impressionistic descriptions by Chao in 1930 are later supported by ample acoustic data^32,34^.

Our first hypothesis was that Cantonese listeners would outperform Mandarin listeners in static musical pitch and interval discrimination. Musical notes typically have stable F0 heights with static F0 movement, and discriminating them relies on perceiving F0 height^24,35^. Cantonese listeners attend to F0 height to distinguish level tone contrasts (55 − 33, 55 − 22, 33 − 22), which mainly differ in F0 height^34^. Contrastively, Mandarin has no level tone contrast, and Mandarin listeners primarily utilize F0 contour but not F0 height for Mandarin tone perception^36,37^. As Cantonese listeners experience a stronger demand for precise F0 height processing than Mandarin listeners^34,36,37^, we hypothesized that the former would discriminate musical pitch and intervals more accurately than the latter.

Our second hypothesis was that Cantonese listeners would also outperform Mandarin listeners in dynamic musical pitch discrimination. When violinists slide the pitch from one musical note to another (i.e., portamento technique), they produce a dynamic F0 contour^38^. Both Cantonese and Mandarin use F0 contour to discriminate contour tone contrasts, but acoustic data showed that Cantonese involves more fine-grained F0 contour variations than Mandarin^32,39^. For instance, Cantonese tones 25 and 23 both have a rising F0 contour, differing only in the magnitude of the F0 slope toward the end of the vocalic segment (see Fig. 1 of [34]). Moreover, Cantonese tones 22 and 21 only differ in the slight F0 fall towards the end of tone 21^34,40^. By contrast, each Mandarin tone has a distinctive F0 contour (see Fig. 1)^32^. Based on the bi-directional OPERA, we predicted that Cantonese listeners would discriminate dynamic musical pitch more accurately than relative to Mandarin listeners.

A previous study offered some preliminary support for our first hypothesis^30^. Dong is a language spoken mostly by an ethnic group in Southwestern China. Relative to Mandarin, Dong has a more complex lexical tone system with nine lexical tones^30^. In their static F0 discrimination task, Dong listeners showed a significantly lower discrimination threshold than Mandarin listeners^30^. Thus, the authors concluded that speaking a more complex tone language would facilitate auditory F0 perception to a larger extent than speaking one with a less complex tone language. That said, the previous study involved pure tones and harmonic tones but not musical pitch, so their finding might not precisely reflect language-to-music transfer. Regarding our second hypothesis, the previous study has not investigated dynamic pitch discrimination. Therefore, the research gap remains and addressing it can provide a nuanced understanding of language-to-music transfer.

Besides accuracy, we measured response time to obtain additional information. In some music-to-language transfer studies, musicians were found to have faster lexical tone perception than non-musicians^19,41–43^, although this was not reported in many other studies^6,7,17,20,21,44^. Pertinent to our focus, none of the language-to-music transfer studies have reported findings related to response time^9–11,24,25,27,28^. Therefore, our hypotheses focus on accuracy and we keep an open mind about whether they also apply to response time.

In short, we aimed to investigate the comparative effects of Cantonese and Mandarin language backgrounds on musical pitch perception. To this end, we examined whether Cantonese listeners outperformed Mandarin listeners in discriminating static musical pitch, musical pitch intervals, and dynamic musical pitch.

## Methods

### Participants

Participants were recruited in Hong Kong through posters, emails, and social media. In Hong Kong, Cantonese is the predominant language used in society, and a small amount of Mandarin instruction is compulsory in primary schools^45^. Therefore, it was challenging to recruit native Cantonese and Mandarin speakers in Hong Kong who were totally unfamiliar with the other language. To tackle this issue as far as practical, our participant recruitment specifically targeted native Cantonese speakers with limited Mandarin proficiency and native Mandarin speakers with limited Cantonese proficiency.

When signing up for the study, all potential participants were screened for their language and music background^46^. In an online questionnaire, they self-evaluated their understanding and fluency of Cantonese and Mandarin on a Likert scale from 1 (unable to understand or speak any words) to 5 (able to understand everything and speak fluently in all situations). To be included in the study, participants had to be fully proficient in their native language (i.e., 5 rating) and non-proficient in the other language (i.e., 1 − 2 rating). Ten participants were excluded as they rated 3 in the other language. Since music training may improve musical pitch perception, we further excluded 12 participants who had received formal music training^47^. The remaining participants had never received any music training besides the typical classroom-based mandatory music lessons at primary and early secondary schools^48,49^.

Our final sample consisted of 24 native Cantonese listeners (11 males, 13 females) and 24 native Mandarin listeners (11 males, 13 females). Their mean ages were 23.62 years (*SD* = 3.44 years) and 23.46 years (*SD* = 3.28 years) respectively. The self-rated Cantonese and Mandarin proficiencies were 5.00 (*SD* = 0.00) and 1.62 (*SD* = 0.51) for the Cantonese listeners and 1.62 (*SD* = 0.51) and 5.00 (*SD* = 0.00) for the Mandarin listeners respectively. In essence, the participants closely matched on their bilingual proficiencies, i.e., being proficient in their native language but not in their non-native language.

### Procedure

This study was ethically approved by the Faculty Research Ethics Committee of the University of Hong Kong. After providing informed written consents, participants completed the static musical pitch, musical pitch interval, and violin glide discrimination tasks in a sound booth at The University of Hong Kong. The tasks were run on E-Prime 3.0 on a laptop computer. The auditory stimuli were presented via Sennheiser HD280 PRO headphones. The intensities of the static pitch and pitch interval stimuli were 93dB while those of the violin stimuli were 78dB. At the beginning of each task, participants were allowed to adjust the volume of the computer such that they would hear clearly at a comfortable level without excessive loudness. Participants were allowed to take a recess at their own pace after each task.

### Static musical pitch discrimination task

#### Stimuli

 The stimuli set contained one reference musical note and 12 altered musical notes^35^. The reference musical note was a computer-generated musical note C4 (261.60 Hz). Based on the C4, six musical notes were created by increasing the F0 by 7, 13, 25, 50, 100, and 200 cents. The six other musical notes were created by decreasing the F0 of the C4 note at the same intervals as above. The duration of each musical note was 600 ms.

#### Stimuli presentation

**Fig. 2:**
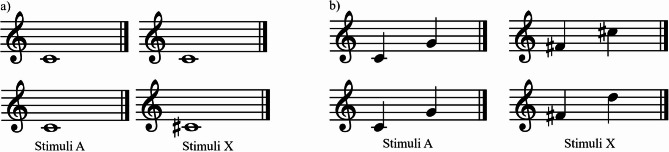
Musical notations of the, stimuli in the (**a**) static musical pitch discrimination and (**b**) musical pitch interval discrimination tasks. The upper rows of both (**a,b**) represent the standard stimuli pair, and the lower rows represent the altered pair.

 This task adopted an AX discrimination paradigm^35^. Each trial contained the musical note C4 followed either by the same musical note or an altered one (see Fig. 2a). The inter-stimulus interval (ISI) was 600ms, allowing sufficient time for pitch judgement^50^. Participants were asked to judge as quickly as possible whether the two musical notes were identical or different. There were two practice trials with feedback on accuracy and 144 experimental trials without feedback (12 AA trials × 6 repetitions + 6 pitch changes × 2 pitch change directions × 6 repetitions). On each trial, the accuracy and response time were recorded. The internal consistency of the task was very high (Cronbach’s alpha = 0.90).

### Musical pitch interval discrimination task

#### Stimuli

 There were one reference stimulus and 12 target stimuli. The reference stimulus was composed of two musical notes, C4 − G4, with a 700-cent interval change. The target stimuli were F#4 followed by a second musical note. The second musical note was either C#5 with a 700-cent interval change relative to F#4, or an altered musical note that was 7, 13, 25, 50, 100 and 200 cents above or below C#5.

#### Stimuli presentation

 This task adopted an AX discrimination paradigm^35^. Each trial contained a reference stimulus C4 − G4 and a target stimulus (see Fig. 2b). On AA trials, the target stimulus was F#4 − C#5, representing the same interval change as C4 − G4. On AB trials, the target stimulus was F#4 and a musical note that was 7, 13, 25, 50, 100 and 200 cents above or below C#5. Participants then judged as quickly as possible whether the reference and target stimuli had the same or different interval changes. There were nine practice trials and 144 experimental trials (12 identical trials × 6 repetitions + 6 pitch changes × 2 pitch change directions × 6 repetitions) were included. Trial accuracy and response time were recorded. The internal consistency of the task was very high (Cronbach’s alpha = 0.90).

### Violin glide discrimination task

#### Stimuli

 Created in a previous study, the stimuli set contained five violin glides^51^. A professional violinist listened to speech recordings of the five Thai tones (high: H, mid: M, low: L, rising: R, and falling: F) and reproduced them with a violin. The F0 ranged between 293 Hz and 456 Hz (i.e., between D4 and A4). Although the violin stimuli took reference of Thai tones, their F0 contour are not exactly the same as those in the speech recordings. Moreover, the stimuli are recognizable as violin sounds instead of human speech^51^.

In total, there were 10 pairs of violin glide contrasts, including three static (M − L, M − H, and L − H) and seven dynamic contrasts (M − R, M − F, L − R, L − F, H − R, H − F, and R − F)^51^. Although our second hypothesis focuses on the seven dynamic contrasts, three static contrasts were included to balance the number of occurrences of each violin glide. It could also offer an extra test for our first hypothesis.

#### Stimuli presentation

 This task utilized the AX discrimination paradigm. On each trial, participants heard two violin glides and then judged as quickly as possible whether they were identical or different. There were nine practice trials and 80 experimental trials (5 identical trials × 8 repetitions + 10 musical pitch contrasts × 4 repetitions). Trial accuracy and response time were recorded. The internal consistency of the task was satisfactory (Cronbach’s alpha = 0.70).

## Results

### Sensitivity index analysis

**Fig. 3:**
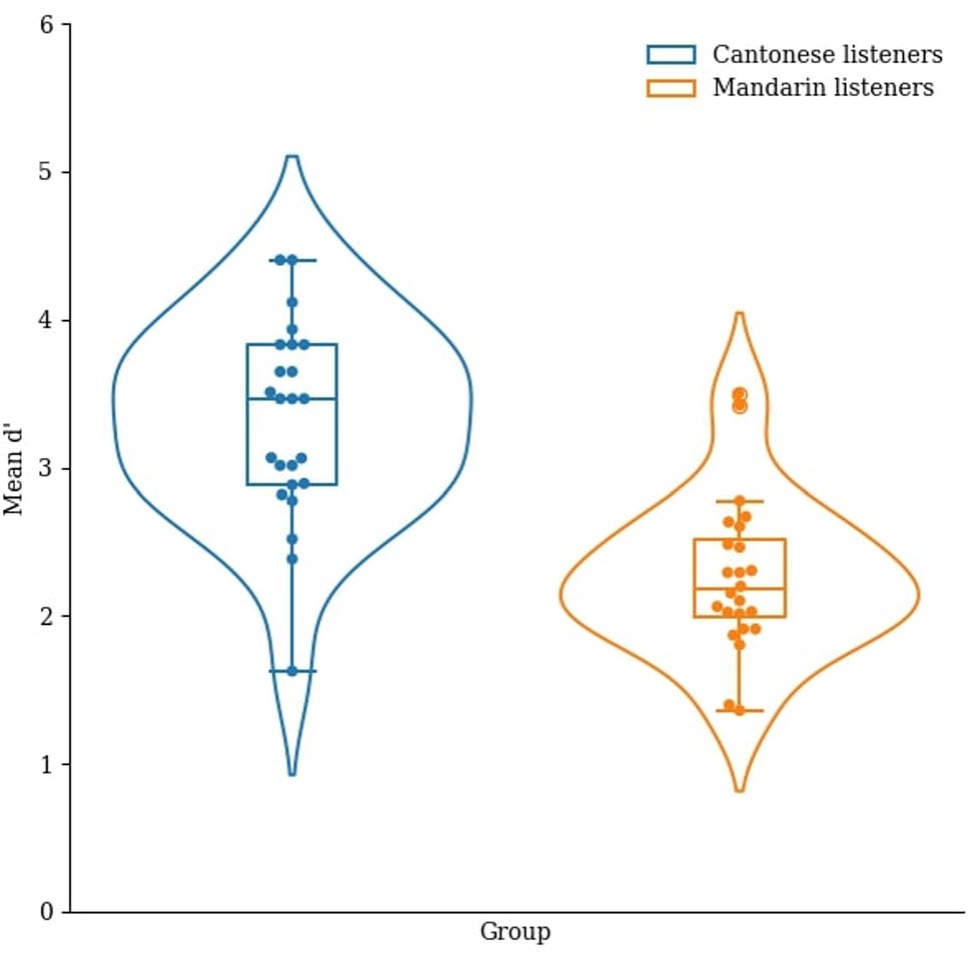
Mean d’ of the Cantonese and Mandarin listeners in the static musical pitch discrimination task.

**Fig. 4:**
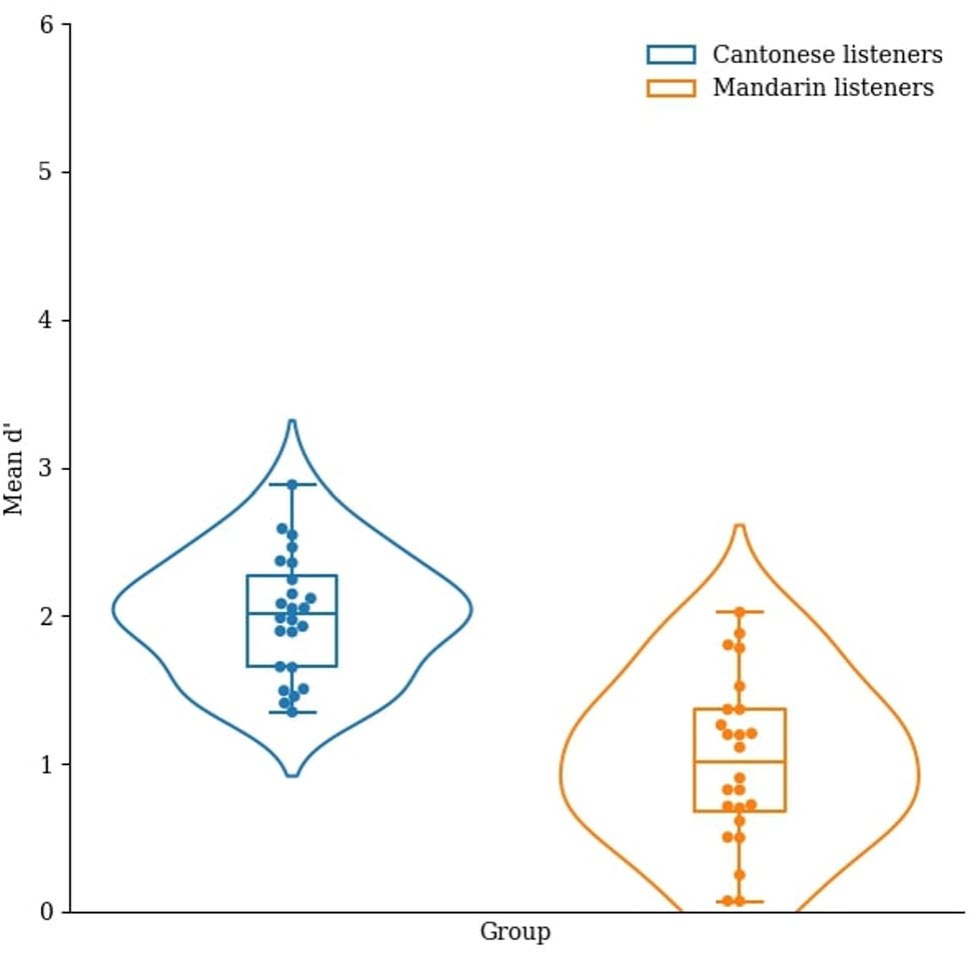
Mean d’ of the Cantonese and Mandarin listeners in the musical pitch interval discrimination task.

**Fig. 5:**
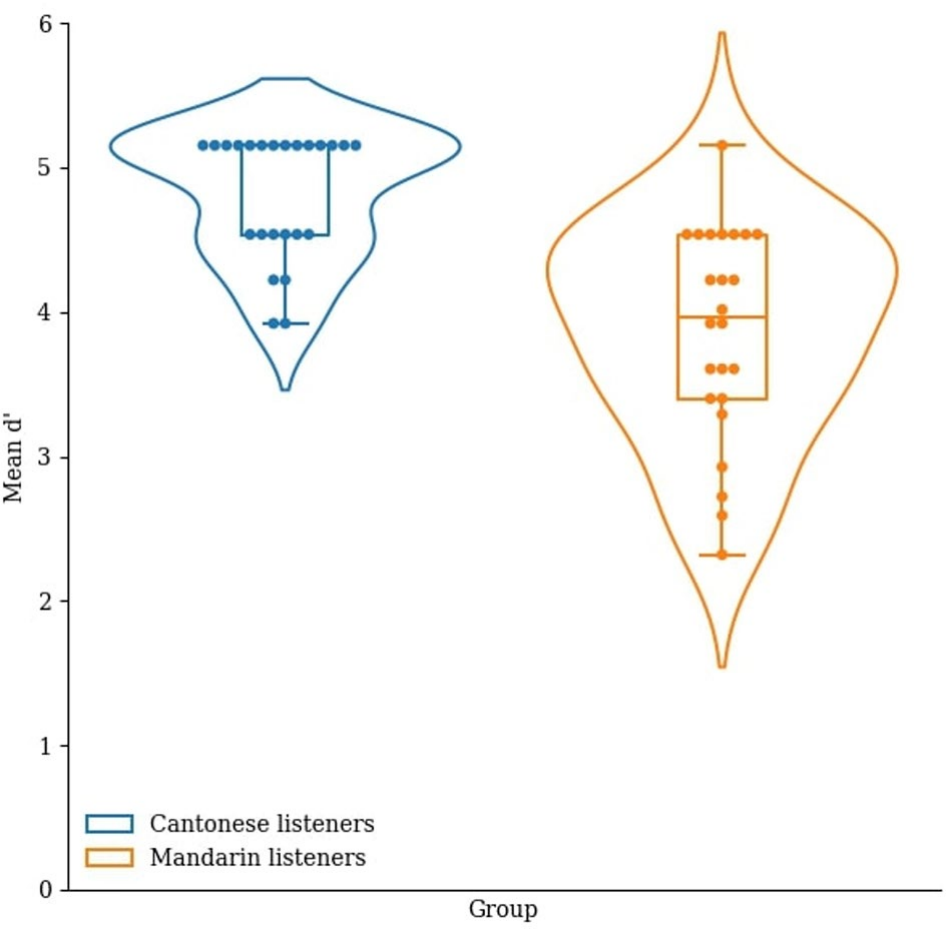
Mean d’ of the Cantonese and Mandarin listeners in the violin glide discrimination task.

Based on the signal detection theory, we calculated the sensitivity index (d’) in the three tasks^52^. Hit rate was defined as the percentage of correctly answered AB trials and false alarm was defined as the percentage of incorrectly answered AA trials. In each task, we obtained the d’ by subtracting the z-transform of the hit rate by that of the false alarm rate (see Figs. 3, 4, 5).

We conducted a one-way MANOΛV with the dependent variables being the d’ in the static musical pitch, musical pitch interval, and violin glide discrimination tasks, and the between-subjects factor being group (Cantonese and Mandarin). Overall, the main effect of group was significant, ʌ_Wilks’_ = 0.34, *F*(3, 44) = 28.16, *p* < .001, η_p_^2^ = 0.66. In particular, the Cantonese listeners had a significantly higher d’ than the Mandarin listeners in the static musical pitch discrimination task, *F*(1, 46) = 38.02, *p* < .001, η_p_^2^ = 0.45, the musical pitch interval discrimination task, *F*(1, 46) = 49.25, *p* < .001, η_p_^2^ = 0.52, and the violin glide discrimination task, *F*(1, 46) = 29.59, *p* < .001, η_p_^2^ = 0.39.

### Mean accuracy analysis

**Fig. 6:**
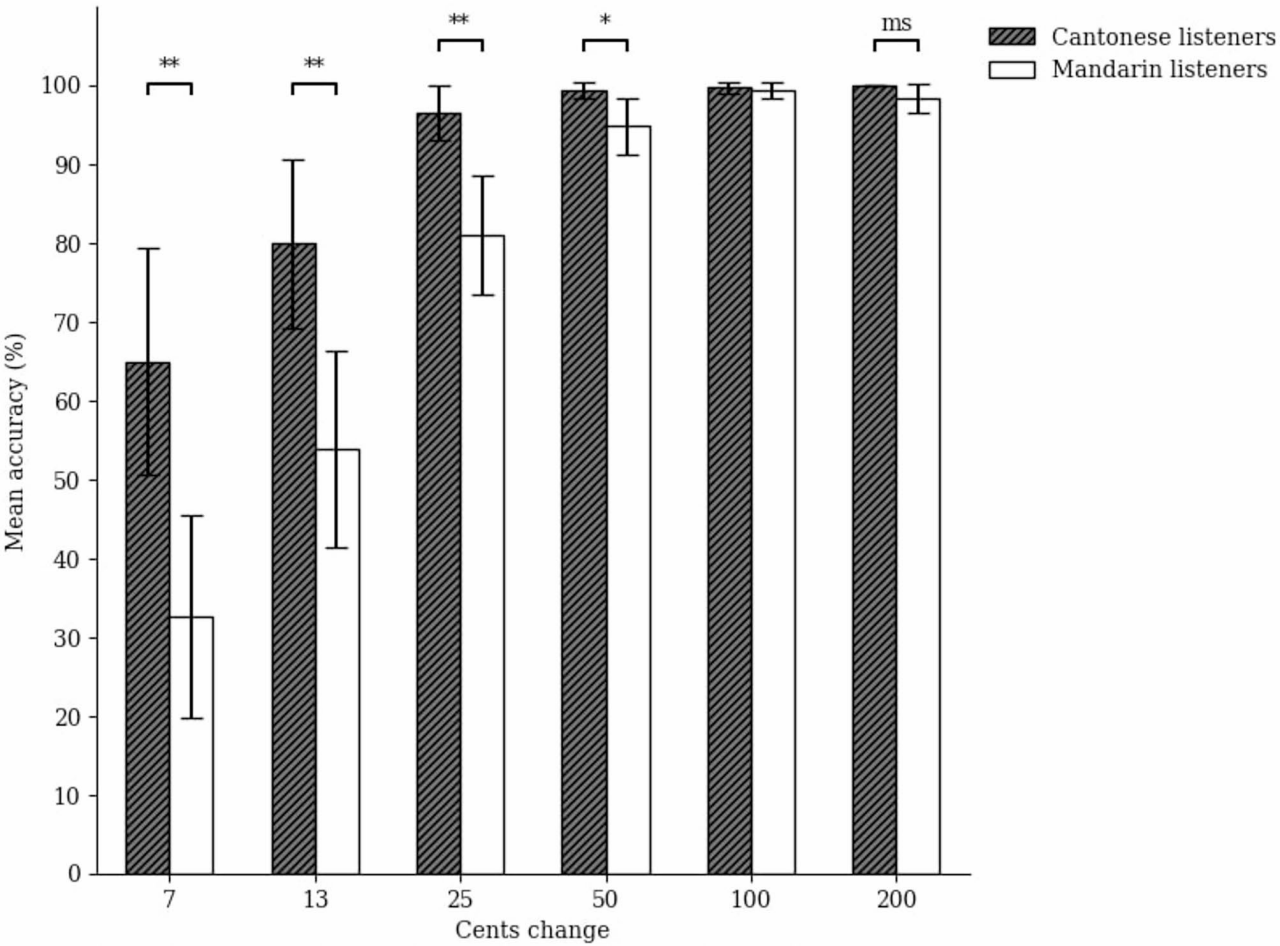
Mean accuracy of the Cantonese and Mandarin listeners across different cents changes in the static musical pitch discrimination task. The error bars represent a confidence interval around the population mean with 0.95 confidence level; *p < .05, **p < .01, ***p < .001, ms marginally significant *p* = .051.

For the static musical pitch discrimination task, we conducted a two-way ANOVA on mean accuracy with cents change (7, 13, 25, 50, 100, and 200) as the within-subject factor, and group (Cantonese and Mandarin) as the between-subjects factor (see Fig. 6). To adjust for the violation of the sphericity assumption, we applied the Greenhouse-Geisser correction to the degree of freedom. The main effect of group was significant, *F*(1, 46) = 18.22, *p* < .001, η_p_^2^ = 0.28, and so was the main effect of cents change, *F*(1.73, 79.56) = 79.71, *p* < .001, η_p_^2^ = 0.63. Critically, the interaction between cents change and group was significant, *F*(1.73, 79.56) = 8.24, *p* = .001, η_p_^2^ = 0.15. Simple effects analysis with Bonferroni corrections revealed that the Cantonese listeners outperformed the Mandarin listeners in discriminating 7, 13, 25, and 50 cents changes, *ps* < 0.05, and marginally in 200 cents changes, *p* = .051, but not 100 cents changes, *p* = .585.

**Fig. 7:**
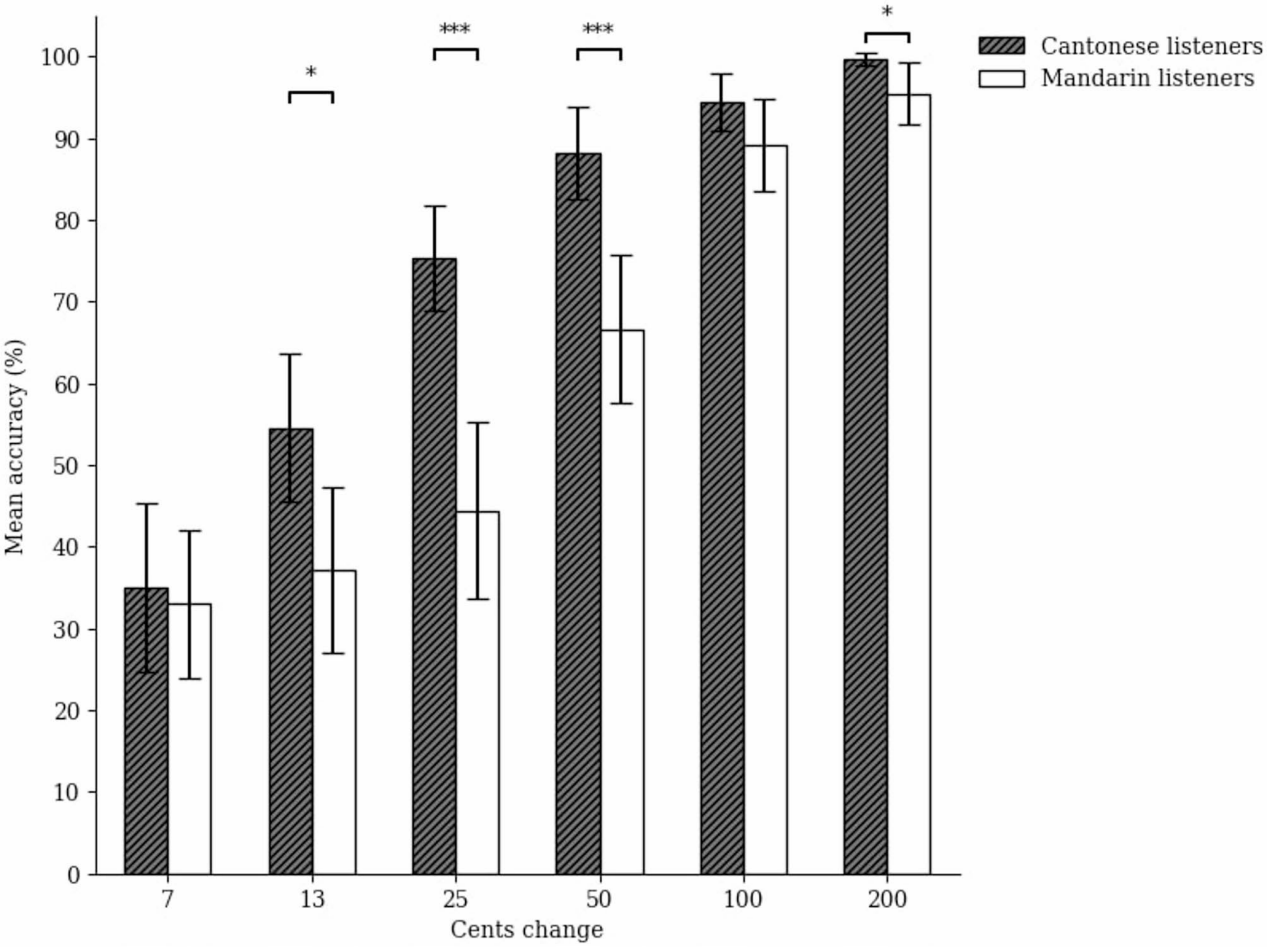
Mean accuracy of the Cantonese and Mandarin listeners across different cents changes in the musical pitch interval discrimination task. The error bars represent a confidence interval around the population mean with 0.95 confidence level; *p < .05, **p < .01, ***p < .001.

For the musical pitch interval discrimination task, we conducted a two-way ANOVA with similar specifications as above (see Fig. 7). There were significant main effects of group, *F*(1, 46) = 18.38, *p* < .001, η_p_^2^ = 0.29, and cents change, *F*(3.29, 151.50) = 123.57, *p* < .001, η_p_^2^ = 0.73. Moreover, the interaction between cents change and group was significant, *F*(3.29, 151.50) = 6.37, *p* < .001, η_p_^2^ = 0.12. Simple effects analysis with Bonferroni corrections showed that the Cantonese listeners outperformed the Mandarin listeners in discriminating 13, 25, 50, and 200 cents changes, *ps* < 0.05, but not 7 and 100 cents changes, *p*s = 0.756 and 0.112 respectively.

**Fig. 8:**
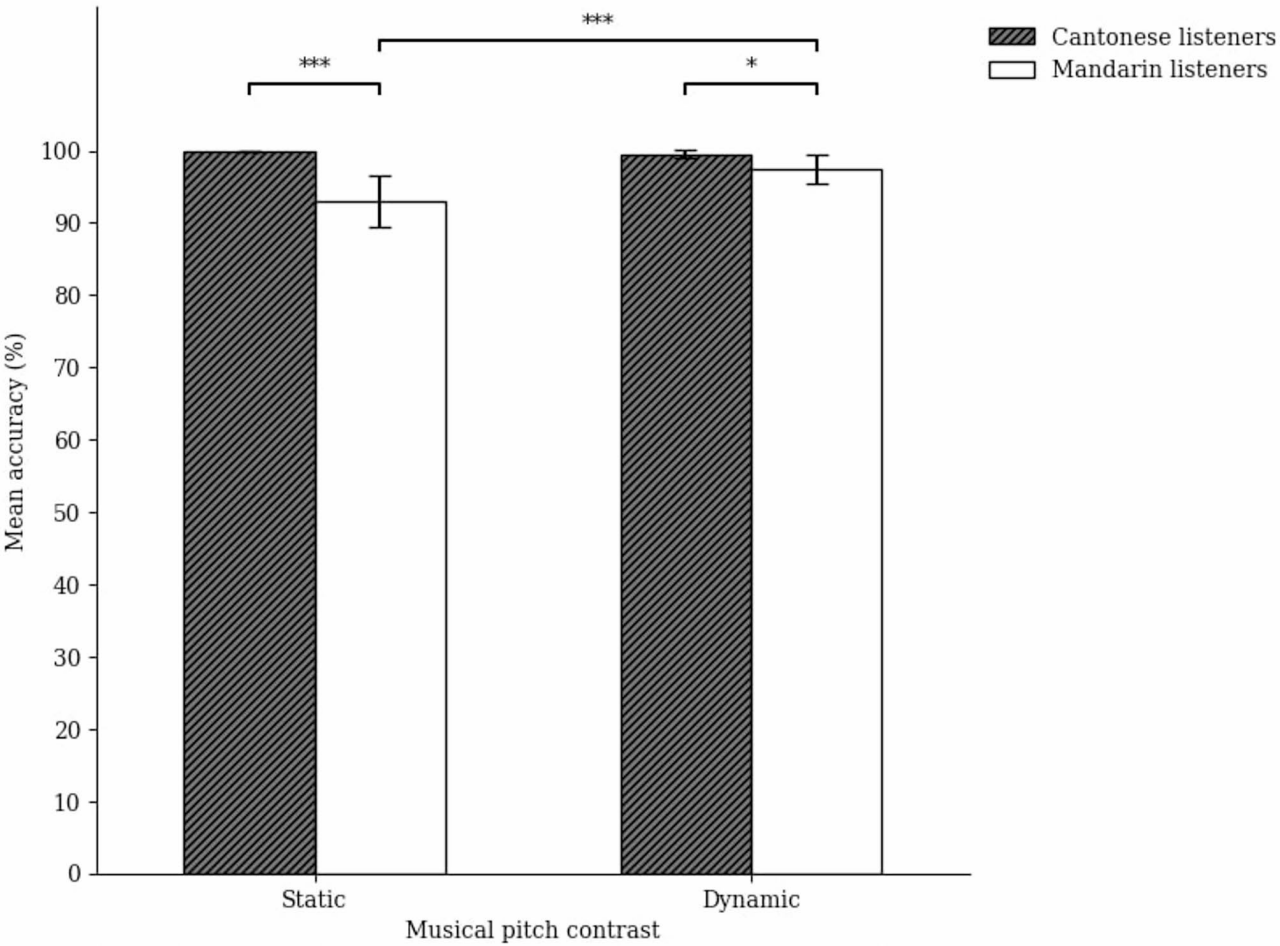
Mean accuracy of the Cantonese and Mandarin listeners across static and dynamic musical pitch contrasts in the violin glide discrimination task. The error bars represent a confidence interval around the population mean with 0.95 confidence level; *p < .05, **p < .01, ***p < .001.

For the violin glide discrimination task, we conducted a two-way ANOVA on mean accuracy with pitch contrast type (static and dynamic) as the within-subject factor and group (Cantonese and Mandarin) as the between-subjects factor (see Fig. 8). There were significant main effects of group, *F*(1, 46) = 14.16, *p* < .001, η_p_^2^ = 0.24, and pitch contrast type, *F*(1, 46) = 7.40, *p* = .009, η_p_^2^ = 0.14. Furthermore, the interaction between pitch contrast type and group was significant, *F*(1, 46) = 11.11, *p* = .002, η_p_^2^ = 0.20. Simple effects analysis with Bonferroni corrections indicated that the Cantonese listeners outperformed the Mandarin listeners in both static, *p* < .001, and dynamic pitch contrasts, *p* = .044. To supplement this finding, we also conducted simple effects analysis at the level of group. Specifically, the Mandarin listeners were significantly more accurate in discriminating dynamic than static pitch contrasts, *p* < .001, whereas the Cantonese listeners showed no significant difference, *p* = .667.

### Response time analysis

For each task, we conducted the same ANOVA as above on the mean response time in the correctly answered trials. Whenever necessary, Greenhouse-Geisser correction to the degree of freedom was applied to adjust for the violation of sphericity assumption. In the static musical pitch discrimination task, the main effect of cents change was significant, *F*(2.37, 94.62) = 10.30, *p* < .001, η_p_^2^ = 0.21, but not the main effect of group, *p* = .941, and the interaction between cents change and group, *p* = .214. Then, post-hoc pairwise comparisons with Bonferroni adjustments were conducted to unpack the main effect of cents change. The mean response time was significantly longer in 7 cents changes than in 25, 50, 100, and 200 cents changes, *ps* < 0.05. Moreover, it was significantly longer in 13 cents changes than in 25, 50, and 200 cents changes, *ps* < 0.05. All other comparisons were not significant, *ps* > 0.05.

In the musical pitch interval discrimination task, the main effect of cents change was significant, *F*(3.22, 125.72) = 8.41, *p* < .001, η_p_^2^ = 0.18, but not the main effect of group, *p* = .659, and the interaction between cents change and group, *p* = .481. The mean response time was significantly longer in 7, 13, and 25 cents changes than in 100 and 200 cents changes, *ps* < 0.05. All other comparisons were not significant, *ps* > 0.05.

In the violin glide discrimination task, the main effect of pitch contrast type was not significant, *p* = .329, nor was the main effect of group, *p* = .502, and the interaction between pitch contrast type and group, *p* = .313.

## Discussion

The present study aimed to investigate the comparative effects of Cantonese and Mandarin language backgrounds on musical pitch perception. We hypothesized that Cantonese listeners would outperform Mandarin listeners in discriminating static musical pitch, musical pitch intervals, and dynamic musical pitch. Our study revealed significant group differences only in d’ and accuracy, not in response time. Therefore, the findings discussed below mainly focus on how accurately, rather than how efficiently, listeners perceive musical pitch.

Consistent with our first hypothesis, Cantonese listeners outperformed Mandarin listeners in the static musical pitch discrimination task. Moreover, Cantonese listeners discriminated static-static violin glides more accurately than Mandarin listeners, providing additional evidence. While previous studies have highlighted the positive impact of a tone language background on static musical pitch perception, our findings suggest that different tone language backgrounds can result in varying degrees of language-to-music transfer^9–11^. As level tone contrasts are only present in Cantonese but not in Mandarin, the former exerts a greater demand on precise F0 height processing than the latter^34,36,37,53–55^. Consequently, Cantonese listeners should be better able to perceive F0 height than Mandarin listeners. As the bi-directional OPERA hypothesis posits, their enhanced neuronal sensitivity to F0 height processing is transferrable to the music domain, enabling them to discriminate static musical pitch more accurately than Mandarin listeners^24^.

Our nuanced analysis revealed that the above finding remained consistent across changes of 7, 13, 25, 50, and 200 cents. Cantonese tones exhibit variations of 200 to 700 cents on average^16^, so language-to-music transfer might be expected only for static musical pitch contrasts with 200-cent changes or above. Contrary to this notion, Cantonese listeners performed better than Mandarin listeners even in discriminating small musical pitch differences of 7 − 50 cents. This finding contradicts the original OPERA hypothesis, which implied that language-to-music transfer could not occur since language requires less precise F0 processing than music^14,15^. It is puzzling why the group differences disappeared at 100 cents but re-emerged at 200 cents. Speculatively, at 100 cents, the task might have entered a boundary zone where the musical pitch difference was perceptually salient and equally clear to both groups, masking the group difference. However, 200-cent changes are within the range of Cantonese tonal variations, so Cantonese listeners might further benefit from linguistic processes^16^. Future studies are needed to examine this speculation or to identify other possible reasons.

In line with our first hypothesis, Cantonese listeners also discriminated static musical pitch intervals more accurately than Mandarin listeners. Extending previous studies, this finding suggested that language-to-music transfer applies to a broader range of pitch processing tasks^11,24,27,28,30^. Specifically, speaking a (complex) tone language may enhance not only the perception of isolated pitch differences, but also the perception of relational pitch information, such as intervals, in musical contexts. Compared with the static musical pitch discrimination results, the only difference is the absence of group difference at 7-cent interval changes. Possibly, 7-cent interval changes might fall within a perceptual boundary where the relational cue (but not the absolute cue) of F0 height may be too subtle. The advantage of Cantonese listeners may only manifest when the relational cue of F0 height is saliant enough to be reliably perceived and utilized, as in 13 cents and above.

Supporting our second hypothesis, Cantonese listeners discriminated dynamic musical pitch more accurately than Mandarin listeners. Compared with the Mandarin tone system, the Cantonese tone system involves more subtle changes in F0 contour^32,34,39,40^. In line with the bi-directional OPERA, Cantonese listeners outperformed Mandarin listeners in discriminating violin glide contrasts involving F0 contour differences. Incidentally, unpacking the pitch contrast type × group interaction revealed that Mandarin listeners found dynamic contrasts easier to perceive compared to static contrasts. This might be because Mandarin listeners primarily attend to F0 contour but not F0 height for Mandarin tone discrimination^53,54^. Contrastively, no such difference was found in the Cantonese listeners, possibly because both F0 height and F0 contour are used for Cantonese tone discrimination^34^.

Alternatively, the findings related to dynamic musical pitch might be attributed to our choice of stimuli. Since the violin glides were modelled after Thai tones, they might not accurately reflect dynamic musical pitch perception. It was possible that, because the Thai tones are already present in Cantonese but not in Mandarin, Cantonese listeners performed better simply due to this familiarity. However, this is unlikely. First, the F0 patterns of the violin glides did not exactly match Thai tones, and the stimuli were clearly recognizable as violin sounds, not speech^51^. Even if they resembled Thai tones, Cantonese and Thai have distinct lexical tone systems, and none of the seven Thai dynamic pitch contrasts exist in Cantonese^34,56^. Additionally, previous research shows Cantonese and Mandarin listeners discriminate Thai tones with similar accuracy^31^. If the stimuli were perceived as Thai tones, both groups should have shown comparable performance, which was not the case.

Incidentally, no significant group differences were observed in response times. To our knowledge, existing studies on language-to-music transfer have similarly not reported any effects on response time^10,11,24,25,27,28^. Collectively, these findings suggest that tone language experience influences how accurately, rather than how efficiently, listeners perceive musical pitch. This may be because processing speed is more reliant on working memory, which is unlikely to be influenced by the type of tone language spoken. While some music-to-language transfer studies—particularly those using mismatch negativity (MMN) measures^41–43^—have reported processing speed advantages, most behavioural research^6,7,17,20,21,44^ has not. Thus, language-to-music transfer (or reverse) may at best enhance neural processing speed without necessarily translating into faster behavioural discrimination.

From a theoretical perspective, the current study offers support for the bi-directional OPERA hypothesis^24^. In this framework, the original domain-relative “precision” component is re-referenced on listeners. As described previously, Cantonese listeners experience a stronger demand on precise F0 height and F0 contour processing than Mandarin listeners. Fitting the prediction of the bi-directional OPERA, Cantonese listeners’ enhanced F0 height and F0 contour processing positively transferred to the musical domain, as evidenced by their superior performance in static and dynamic musical pitch discrimination.

In the broader literature, our study enriches the body of evidence on language-to-music transfer. As previously mentioned, previous studies have compared musical pitch perception between listeners from a tone language background and those who were not^11,24,27^. Our study provided fine-grained evidence suggesting that different tone languages could have varying effects, or at least different degrees of a positive effect, on musical pitch perception. As discussed earlier, the original OPERA hypothesis cannot fully explain the growing evidence of language-to-music transfer^14,15^. The present and previous findings underscore the need to theoretically account for language-to-music transfer. Of course, the bi-directional OPERA is by no means the only possible account^24^. Researchers may want to propose and test some new hypotheses.

There are several limitations and potential avenues to extend the current study. For practical reasons, we were unable to recruit Cantonese and Mandarin listeners who were completely unfamiliar with the other tone language. Future research could investigate language-to-music transfer among monolingual speakers of tone languages. Due to time constraints, we relied solely on self-rated language proficiency. Although the participants closely matched on this variable, it would have been ideal to include objective assessments of language proficiency and perhaps even Cantonese and Mandarin tone discrimination tasks to serve as covariates. Regarding the time scale of musical notes, pitch glides typically involve either rising or falling F0 contours; therefore, our violin glide discrimination task did not include falling-rising or rising-falling contours. These types of contours could be incorporated in future studies that examine musical pitch perception at the melodic level with a larger time scale.

Additionally, with the behavioural evidence now established, future studies could delve into the neural mechanisms underlying the comparative effects of different tone language backgrounds on musical pitch perception. For example, the bi-directional OPERA hypothesis predicts that Cantonese listeners should exhibit the largest MMN amplitude in response to musical pitch violations, followed by Mandarin and then English listeners. It would also be interesting to examine whether language-to-music transfer results in faster processing speed, as indicated by earlier MMN latencies.

In conclusion, Cantonese listeners outperformed Mandarin listeners in musical pitch perception. Consistent with our hypothesis, Cantonese listeners discriminated static musical pitch, musical pitch interval, and dynamic musical pitch more accurately than did Mandarin listeners. While previous studies often treated tone language background as a binary yes-or-no variable, our study suggests that different tone language backgrounds may lead to varying degrees of language-to-music transfer^24,25,28^. From a theoretical perspective, the findings from our study supported the bi-directional OPERA hypothesis^24^. Broadly speaking, both past and present research highlights the need to theoretically account for language-to-music transfer^9,11,27^.

## Data Availability

The datasets generated and analysed during the current study are available in the Open Science Framework repository, https://osf.io/t9ehw/?view_only=4548878d865642f58e24dbee612fdaa5.
